# When helpful becomes harmful: a case-based narrative review of esophageal mesh migration after hiatal hernia repair

**DOI:** 10.20452/wiitm.2025.17982

**Published:** 2025-09-26

**Authors:** Natalia Dowgiałło ‑Gornowicz, Dominika Mysiorska, Eliza Dobruchowska ‑Kęsikowska, Paweł Lech

**Affiliations:** Department of General, Minimally ‑Invasive, and Elderly Surgery, Collegium Medicum, University of Warmia and Mazury, Olsztyn, Poland; Department of Anesthesiology and Intensive Care, Municipal Polyclinical Hospital, Olsztyn, Poland

**Keywords:** hiatal hernia, hiatal hernia repair, mesh migration, Seramesh PA DRUM, sleeve gastrectomy

## Abstract

**INTRODUCTION:**

Hiatal hernia (HH) repair with mesh reinforcement is a commonly performed procedure to reduce the recurrence of HH. Mesh migration (MM) remains a rare but serious complication.

**AIM:**

The aim of this study was to analyze the existing literature on MM after HH repair in the context of a clinical case discussion.

**MATERIALS AND METHODS:**

This study is a nonsystematic narrative review supplemented by a case report.

**RESULT:**

A 70-year-old woman who underwent HHR with partially absorbable Seramesh PA DRUM mesh was diagnosed with MM into the esophagus 9 months postoperatively. The esophageal fistula was treated successfully with endoscopic vacuum therapy. Three-month follow-up showed stable oral intake without symptoms. MM is influenced by mesh material, fixation technique, and esophageal dynamics. Clinical presentation often includes dysphagia, pain, and weight loss, while diagnosis relies on endoscopy imaging. Management strategies vary from observation to endoscopic or surgical removal, with minimally-invasive approaches preferred when feasible. This case is the first reported instance involving the Seramesh PA DRUM mesh, and highlights the potential role of immune dysregulation and hypersensitivity in MM.

**CONCLUSION:**

Early recognition, individualized management, and the use of minimally-invasive techniques may improve outcomes. Continued research and long-term follow-up are essential to better understand risk factors and establish optimal treatment strategies.

## INTRODUCTION

Hiatal hernia (HH) is a condition in which the gastroesophageal junction—and in severe cases, the stomach or other abdominal organs—protrude through a widened esophageal hiatus into the thoracic cavity.^1^ The presence of HH leads to the development of gastroesophageal reflux disease (GERD), which results in decreased quality of life due to persistent symptoms.^2^;^3^

Laparoscopic antireflux surgery is the standard method for treating GERD, offering excellent long-term outcomes and high patient satisfaction.^4^ However, a significant rate of intrathoracic wrap migration or HH recurrence has been reported.^5^;^6^ HH recurrence rates range from 12% to 42%.^6^

Mesh augmentation is a highly controversial adjunct to HH repair (HHR). However, with the aim of avoiding the disadvantages of both nonresorbable synthetic and biological materials, recently developed biosynthetic long-term resorbable meshes are becoming increasingly popular.^7^ Both biological and long-term resorbable meshes are mainly indicated for large hiatal defects and paraesophageal hernias, where primary suture repair alone carries a higher risk of recurrence.^7^

Biological mesh offers a temporary collagen scaffold that reduces short-term recurrence without mesh-related complications, while absorbable synthetic mesh shows promising recurrence rates with no major complications, potentially balancing reinforcement and safety.^8^ Biological meshes are favored by some surgeons for their rapid revascularization and resistance to infection, while long-term resorbable meshes aim to combine these benefits with lower cost and elimination of a permanent foreign body.^9^ The use of bioabsorbable meshes can reduce the HH recurrence rate from 14% to 2% with similar operative time and length of hospital stay.^10^

Nevertheless, using mesh to reinforce cruroplasty remains controversial due to limited evidence of its benefit and potential complications, such as dysphagia and esophageal erosion. Current guidelines recommend mesh only in selected patients with weak crura or large hiatal defects.^11^ Moreover, mesh selection remains controversial due to limited high-quality comparative data, and the decision to use mesh should be individualized.^6^

Due to a growing interest in the use of surgical meshes and emergence of new companies manufacturing them, it is important to raise awareness among surgeons about the potential rare complications that may occur following mesh implantation.

## AIM

The aim of this study was to analyze the existing literature on mesh migration (MM) after HHR in the context of a clinical case discussion.

## MATERIALS AND METHODS

This study is a nonsystematic narrative review supplemented by a case report. The literature review was conducted using the PubMed database in August 2025. Keywords included “mesh erosion,” “hernia mesh complications,” and “mesh migration.” Inclusion criteria were: availability of full-text articles, English language, and publication types, such as case reports, case series, and review articles. Exclusion criteria involved studies not published in English or those without full-text availability.

The patient described in the case report was treated at the Department of Surgery of the Municipal Hospital in Olsztyn, where all her surgical procedures were performed between 2023 and 2025. She provided written informed consent for the analysis and publication of her case. The study was conducted in accordance with the principles of the Declaration of Helsinki, and all ethical standards for patient data confidentiality and case reporting were maintained.

## RESULTS

### Case presentation

The patient was a 70-year-old woman with a history of sleeve gastrectomy performed in July 2023. After the surgery, she developed symptoms of severe GERD, with a radiologically confirmed sliding HH measuring 66 mm × 55 mm. Due to acceptable weight loss and improvement of obesity-related diseases, and with the patient’s consent, it was decided to perform HHR with mesh placement in July 2024. First, the distal esophagus was dissected and brought down 4 cm below the esophageal hiatus. HHR was performed by suturing the posterior crura of the diaphragm using a nonabsorbable barbed suture. The mesh used was a 2-piece 6 cm × 6 cm polypropylene (PP) / polyglycolic acid-caprolactone, undyed, monofilament, partially absorbable mesh Seramesh PA DRUM (Serag-Weissner, Naila, Germany). The mesh was fixed with a Glutack device (GEM, Viareggio, Italy).

The patient’s medical history included type 2 diabetes mellitus (T2DM), arterial hypertension, prior total hip arthroplasty, and multiple joint dislocations. She was allergic to: paracetamol, metamizole, acetylsalicylic acid derivatives, furazidin, morphine, and fosfomycin. Her maximum body mass index (BMI) was 40 kg/m². At the time of the HHR, her BMI had decreased to 29 kg/m², accompanied by complete remission of T2DM and improved control of hypertension.

**FIGURE 1:**
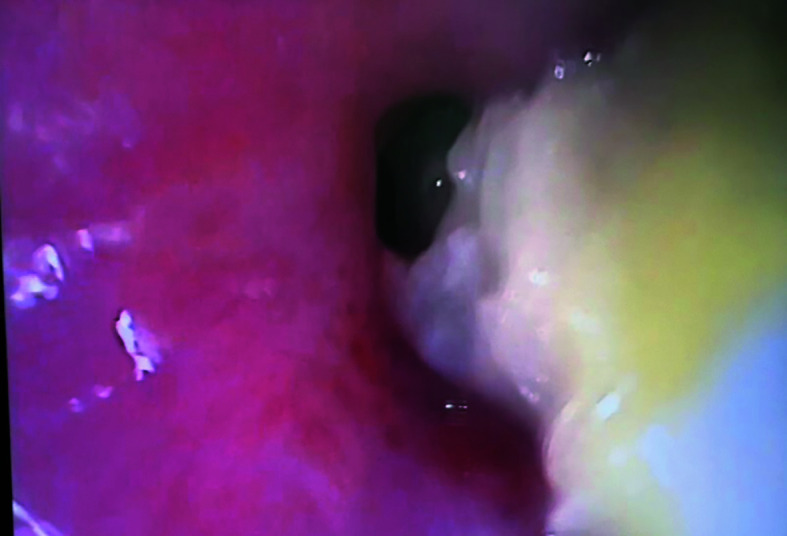
Gastroscopy showing a stent mesh in the lower part of the esophagus

**FIGURE 2:**
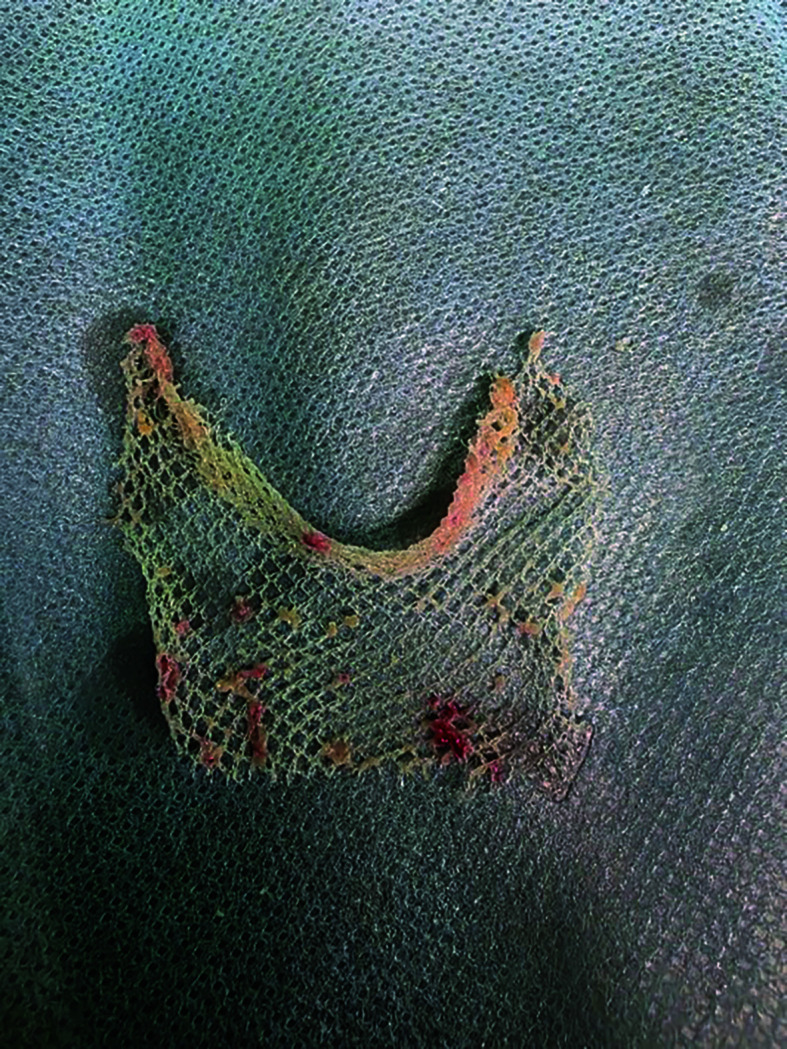
Mesh removed via endoscopy

Five months after the HHR surgery, the patient began experiencing progressive postprandial abdominal pain and recurrent urinary tract infections. In April 2025, she was admitted to a hospital, where esophagogastroduodenoscopy showed esophageal fistula caused by MM into the esophagus FIGURE 1. The mesh was removed endoscopically FIGURE 2. Then, a fully covered self-expanding Niti-S MEGA Esophageal Stent (TaeWong, Gimpo-si, South Korea) was placed.

**FIGURE 3:**
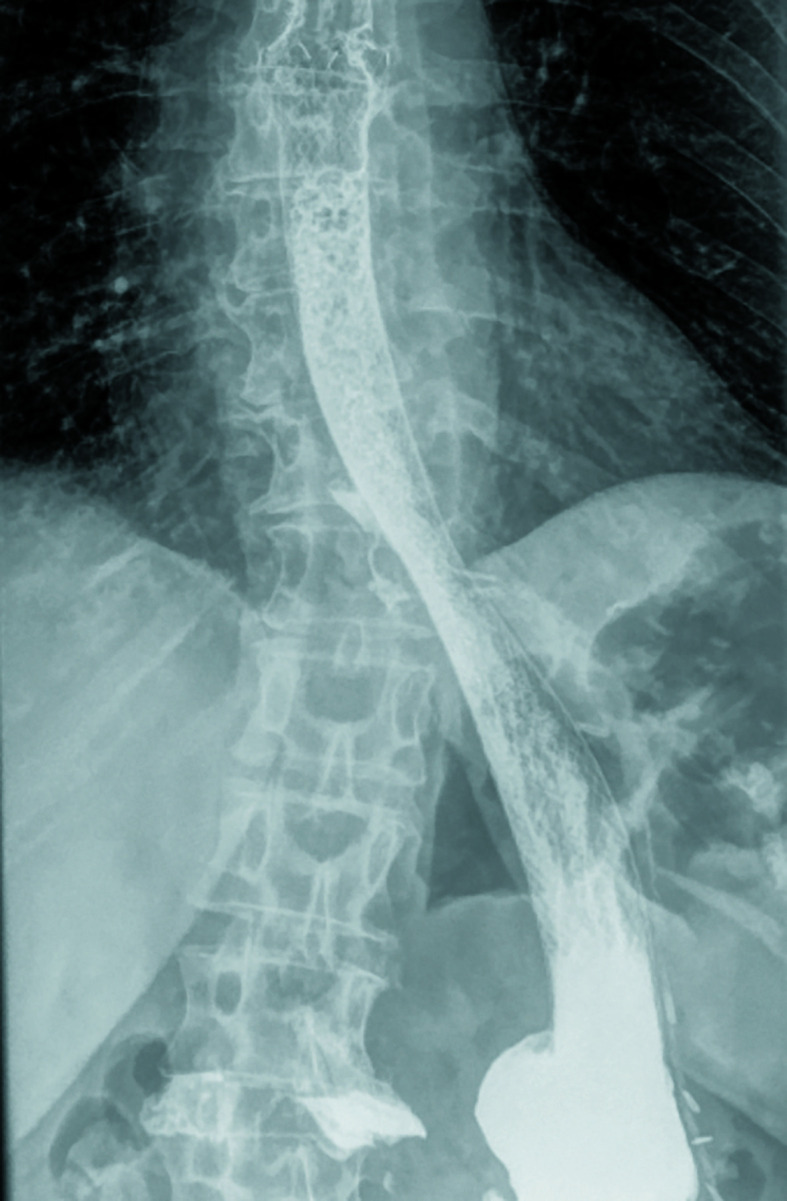
Contrast X‑ray of an esophagogastric stent placed, with the arrow indicating the site of leakage

Two weeks later, symptomatic leakage recurred FIGURE 3. The stent was removed and replaced with the VAC Stent GI system (Micro-Tech, Düsseldorf, Germany) for 5 days. The vacuum-assisted closure system significantly reduced the size of the fistula. Subsequently, self-made custom stents were used 2 times in 4 days, leading to further improvement.

After 2 months of treatment, the patient was discharged home with a nasojejunal feeding tube (Flocare Bengmark NI Tube, Nutricia, Wuxi, China) for nutritional support, which was removed 1 month later. The patient was in a stable condition and was eating orally without complaints at 3-month follow-up.

### Epidemiology of mesh migration

The true epidemiology of MM is not precisely known, as it is a rare complication, and not all cases have been reported in the literature. In their systematic review, ^12^ reported the incidence of mesh-related complications to be 0.035%, based on the observational studies they analyzed. Statistically, this means that approximately 3000 HHRs would have to be performed to observe 1 case of MM.

MM can occur as early as several days after surgery or even many years later. The longest interval found in the literature was a case of PP MM into the stomach 20 years after Nissen fundoplication in a woman who was 19 years old at the time of surgery.^13^;^14^ reported a case of esophageal MM just 11 days after surgery. It involved a 59-year-old woman who underwent emergency Nissen fundoplication due to gastric volvulus.^14^;^15^ described a case of MM 4 occurring months after the repair of a recurrent paraesophageal hernia using a composite polytetrafluoroethylene / expanded polytetrafluoroethylene (PTFE/ePTFE) prosthesis in an 80-year-old woman. Other studies have reported early (<⁠6 months) MM in patients over the age of 65 years.^16^;^17^;^18^;^19^;^20^ This, however, should not be considered a rule, but rather an observable trend in the literature.

### Pathogenesis of mesh migration

^21^ reported that MM occurs in approximately 50% of cases in the esophagus, 25% in the stomach, and 23% at the gastroesophageal junction. The esophagus is a mobile organ that is directly affected by the dynamic movements of respiration and swallowing, which can lead to mechanical damage, friction, and microtrauma—factors that can easily contribute to MM.^22^

In clinical practice, several types of mesh materials can be used. There are 2 main types of nonabsorbable materials: synthetic and biological.^23^ PP is a nonabsorbable polymer with considerable tensile strength. It has a highly fibrogenic nature, inducing a strong inflammatory response and fibrosis, which increases the risk of esophageal wall penetration and stricture formation.^12^;^21^;^24^ PTFE and ePTFE are inert materials characterized by low reactivity and reduced inflammatory response.^23^ While their use minimizes adhesion formation, poor tissue integration may lead to encapsulation of the implant and an increased risk of migration. Most cases described in the literature involve MM with the use of this type of meshes. However, it results not only from their compliant structure, but also from the fact that they have so far been the most commonly used meshes in HHR, which is why most data available refer to them.^12^

Composite meshes (eg, PP coated with a resorbable anti-adhesive layer) are designed to limit contact between the mesh and visceral organs, thereby reducing the formation of adhesions.^23^ Biological meshes derived from processed human or animal tissues offer the highest level of biocompatibility and carry a minimal risk of migration or erosion.^23^ Due to their natural incorporation into host tissue, they are considered safe even in contaminated surgical fields. However, their main drawback is lower mechanical durability and a higher hernia recurrence rate.^25^

In summary, MM may be caused by its placement near the esophagus. Since meshes tend to contract, this can lead to displacement or pressure on the adjacent tissues. Other causes comprise improper fixation techniques, including insufficient or excessive immobilization and a lack of appropriate distance from the esophagus. Additionally, esophageal movements can exacerbate the process. The presence of a foreign body induces an intense inflammatory response, which can, through necrosis and tissue dissolution, lead to MM.

### Clinical symptoms and diagnosis of mesh migration

The main symptoms of MM described in the literature are progressive dysphagia and increasing epigastric pain.^12^ This results from the reaction of the esophagus to mesh erosion. Passing food causes movement of the mesh and pain, as well as a direct inflammatory response in the surrounding tissues. Due to reduced food intake, patients often experience weight loss, which, combined with dysphagia, may raise oncologic suspicion.^18^;^26^;^27^;^18^ reported a case of achalasia caused by mesh erosion, presenting with nocturnal cough and a 16-kg weight loss. ^28^ described an interesting case of a patient presenting with severe persistent halitosis attributable to mesh erosion, occurring 8 years after redo laparoscopic HHR.

Since symptoms, especially dysphagia, often occur after uncomplicated HHRs even without the use of mesh, they can be easily overlooked initially.^2^ If full or partial MM has occurred, it can be detected during esophagogastroduodenoscopy. In the cases where the mesh causes a leak, a fistula may be visible on contrast radiographic studies. Computed tomography is a useful tool for assessing the extent of the disease and inflammatory changes rather than diagnosing mesh erosion itself.

### Management of mesh migration

Due to the rare occurrence of this complication, there are no clear guidelines regarding MM management. The approaches described in the literature so far have considered both the general condition of the patient and the experience of the treatment center, which appears to be a reasonable strategy.

^21^ demonstrated that 10 out of 15 cases (19.6%) were managed with surgical mesh removal, and the same number underwent distal esophagectomy. In 15.7% of the patients analyzed, the mesh was successfully removed endoscopically. ^29^ reported the use of a stent in 1 patient with MM; however, no details were provided regarding the rationale for this approach. ^30^ reported a series of MMs in their institution. Among the 6 patients described, the mesh was left in place in 2 asymptomatic individuals, and a decision was made to pursue observation and conservative management.

Based on the currently available data, no single management strategy can be considered definitively safe or universally appropriate, and treatment decisions should be made on a case-by-case basis. Whenever possible, minimally-invasive techniques should be prioritized. In asymptomatic patients, leaving the mesh in place with close monitoring can be considered a viable option. When endoscopic removal is feasible, this approach should be preferred over extensive surgical intervention. Conventional surgical procedures should be reserved for cases where minimally-invasive methods have failed or are not applicable.

## DISCUSSION

To the best of our knowledge, our manuscript presents the first reported case of mesh erosion involving Seramesh PA DRUM. It is a partially absorbable mesh composed of PP and polyglycolic acid-caprolactone. The combination of PP and a biologically absorbable component is theoretically intended to balance the risk of MM with the risk of hernia recurrence. However, current literature demonstrates that no mesh material is entirely free from the risk of complications, and no type guarantees complete avoidance of adverse outcomes.^12^;^21^

In our case, the patient was over 70 years old and had a history of multiple drug allergies. It may indicate an underlying immune dysregulation or hypersensitivity phenotype. Aging is associated with immunosenescence, which is a progressive decline in immune system competency, paired paradoxically with persistent low-grade inflammation.^31^;^32^ At the same time, patients with multiple drug allergies may display enhanced immune hyperreactivity resulting in exaggerated hypersensitivity reactions, even in response to biologically inert materials. Combined reactions may have caused a dysregulation of the immune response to the mesh, resulting in excessive inflammation and fibrotic remodeling, as well as leading to esophageal erosion and, consequently, MM. Studies show that in both immunocompromised individuals and elderly patients, antireflux surgery and mesh placement can be performed safely.^33^;^34^ However, the increased risk of complications must always be considered.

The application of endoscopic vacuum therapy (EVT) represents a novel and increasingly recognized strategy for managing esophageal fistulas secondary to MM. Although still relatively new, EVT is being reported more frequently and used in various gastrointestinal tract fistulas.^35^;^36^;^35^ demonstrated that EVT is a safe and effective treatment modality for leaks and fistulas following metabolic and bariatric surgery. The application of negative pressure helps reduce fistula size and promotes granulation tissue formation and healing. Potentially, EVT reduces the need for complex reoperations or esophageal resections, which were commonly required in earlier case series.^12^;^21^

In cases of MM, it is also important to emphasize early initiation of enteral nutrition. This highlights the critical role of multidisciplinary care in the management of complex MM. Currently, there is no consensus or formal guideline regarding the treatment of MM. Given the rarity and complexity of such cases, there is a pressing need for larger multicenter registries and prospective studies to establish evidence-based protocols and improve patient outcomes.

This study has several limitations. It is based on a nonsystematic narrative review, which may introduce selection bias due to the lack of methodological rigor typical of a systematic review or meta-analysis. Secondly, the case report reflects only a single patient’s experience, which limits the generalizability of the findings. While the use of EVT in this case appears to be successful, it remains an emerging technique with limited high-quality data. Further studies are necessary to confirm its efficacy and establish its role in clinical practice. Additionally, our patient was followed up for 3 months. Despite the absence of symptoms and good tolerance of oral intake, it is still important to remain aware of possible further complications.

## CONCLUSIONS

MM is a rare but potentially serious complication following HHR. Early recognition, individualized management, and the use of minimally-invasive techniques may improve outcomes. Continued research and long-term follow-up are essential to better understand risk factors and establish optimal treatment strategies.
